# Genetic Components of 25-Hydroxyvitamin D Increase in Three Randomized Controlled Trials

**DOI:** 10.3390/jcm9020570

**Published:** 2020-02-19

**Authors:** Olivia Trummer, Natascha Schweighofer, Christoph W. Haudum, Christian Trummer, Stefan Pilz, Verena Theiler-Schwetz, Martin H. Keppel, Martin Grübler, Thomas R. Pieber, Wilfried Renner, Barbara Obermayer-Pietsch, Elisabeth Lerchbaum

**Affiliations:** 1 Division of Endocrinology and Diabetology, Department of Internal Medicine, Medical University of Graz, 8036 Graz, Austria; natascha.schweighofer@medunigraz.at (N.S.); christoph.haudum@medunigraz.at (C.W.H.); christian.trummer@medunigraz.at (C.T.); stefan.pilz@medunigraz.at (S.P.); verena.schwetz@medunigraz.at (V.T.-S.); martin.gruebler@gmx.net (M.G.); thomas.pieber@medunigraz.at (T.R.P.); barbara.obermayer@medunigraz.at (B.O.-P.); elisabeth.lerchbaum@medunigraz.at (E.L.); 2 Center for Biomarker Research in Medicine, CBmed, 8010 Graz, Austria; 3 Department of Laboratory Medicine, Paracelsus Medical University Salzburg, 5020 Salzburg, Austria; m.keppel@salk.at; 4 Clinical Institute of Medical and Chemical Laboratory Diagnostics, Medical University of Graz, 8036 Graz, Austria; wilfried.renner@medunigraz.at

**Keywords:** vitamin D, vitamin D supplementation, vitamin D metabolism, genetics

## Abstract

The 25-Hydroxyvitamin D (25[OH)D) serum concentration depends on vitamin D intake, endogenous vitamin D production and genetic factors. The latter have been demonstrated in large genome-wide association studies indicating that single nucleotide polymorphisms (SNPs) in genes related to the vitamin D metabolism are as important for serum 25(OH)D levels as the influence of season. The mechanism on how these SNPs influence serum 25(OH)D levels are still unclear. The aim of the present study was to investigate the genetic effects of ten selected SNPs related to vitamin D metabolism on 25-hydroxyvitamin D increase (∆25(OH)D) after vitamin D supplementation in three randomized controlled trials. Genotypes of SNPs related to vitamin D metabolism were determined in 411 participants with 25(OH)D concentrations < 75 nmol/l receiving 20,000 IU cholecalciferol per week for 8 or 12 weeks after study inclusion. For the vitamin D receptor (VDR) rs10783219 polymorphism, the minor A-allele was associated with lower ∆25(OH)D values in the entire study population (*p* = 0.022), which was not consistent in all three cohorts when analysed separately. VDR rs10783219 might therefore be a genetic modulator of increasing 25-hydroxyvitamin D concentrations. Considering the wide-spread use of vitamin D supplementation, future large and well-designed randomized controlled trials (RCTs) should investigate the clinical impact of this polymorphism.

## 1. Introduction

Vitamin D is known for its fundamental role in calcium and bone metabolism [1,2]. Due to the fact that the vitamin D receptor (VDR) is expressed in almost every tissue and cell throughout the human body, there have been extensive investigations on potential extra-skeletal effects of vitamin D [3]. Observational studies showed that low 25-Hydroxyvitamin D (25[OH)D) concentrations were associated with numerous acute and chronic diseases as well as increased mortality, thus raising a high interest in vitamin D [4,5]. However, randomized controlled trials (RCTs) have largely failed to show significant effects of vitamin D supplementation on various health outcomes [3] and Mendelian randomization studies suggested that low 25(OH)D concentrations in the respective studies might be prone to reversed causality, i.e., being consequences rather than causes for associations like mortality [6] or cancer [7]. Nevertheless, this does not invalidate the potential predictive power of 25(OH)D concentrations for these associations.

Results of twin studies have shown a heritable component of circulating 25(OH)D concentrations, with heritability rates ranging from 29% to 77% [8,9,10]. In large genome-wide association studies (GWAS), it has been reported that single nucleotide polymorphisms (SNPs) in the vitamin D binding protein (group specific component (GC)) as well as in enzymes related to activation or degradation of vitamin D and its metabolites are of similar importance for serum 25(OH)D levels as the effect of season [11,12,13,14] These polymorphisms influence 25(OH)D levels derived from endogenous production via ultraviolet light (UV) exposure as well as the increase of 25(OH)D levels after vitamin D intake by food.

It is plausible that they may also determine the rise of 25(OH)D levels in response to intake of vitamin D supplements. A considerable variability in individual responses to vitamin D supplementation has been demonstrated, further suggesting that a one-size-fits-all approach to vitamin D supplementation may not be appropriate. Other factors like vitamin D supplement dose [15], body weight [16,17], baseline serum 25(OH)D level [18,19] and the season in which supplementation is initiated [20] may also contribute to individual variability of 25(OH)D changes in response to vitamin D supplementation. Although there is strong evidence that vitamin D status is affected by specific genotypes, only a few studies investigated a potential role of genetic polymorphisms on the response to vitamin D supplementation [21,22,23].

In the present study, we aimed to investigate associations of ten selected polymorphisms out of six genes related to vitamin D metabolism with the increase of 25(OH)D levels (∆25(OH)D) after vitamin D supplementation in three different RCTs.

**Figure 1:**
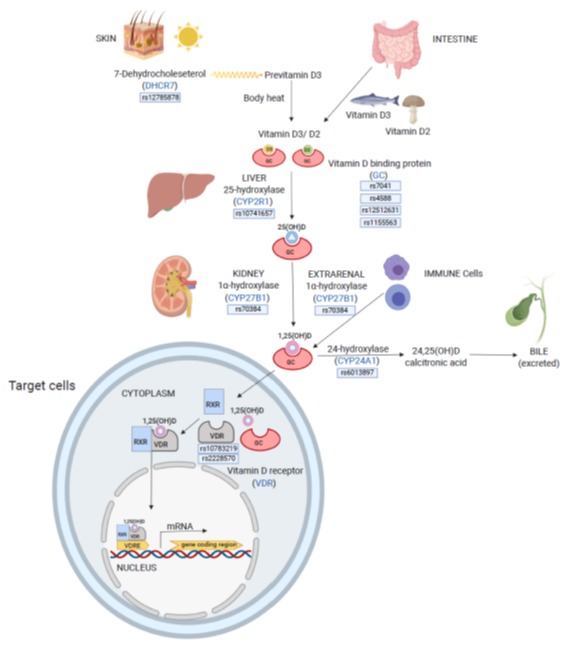
Selected genes related to vitamin D metabolism: Vitamin D (ergocalciferol (vitamin D2) and cholecalciferol (vitamin D3)) can be obtained from foods or endogen synthesis (vitamin D3) in the skin. During cholesterol synthesis, 7-Dehydrocholesterol is formed by the enzyme 7-dehydrocholesterolreductase (DHCR7) as an intermediate product that is converted to vitamin D3 on exposure to sunlight. Vitamin D2, D3 and their metabolites are transported into the blood by vitamin D binding proteins (GC (group-specific components)). Vitamin D is biologically inactive, and its activation requires two enzymatic reactions: the first step is 25-hydroxylation by the hepatic enzyme 25-hydroxylase (CYP2R1), which produces 25-hydroxyvitamin D (25(OH)D) that is the major circulating form of vitamin D. The second step is 1α-hydroxylation by the enzyme 1α-hydroxylase (CYP27B1), which produces the biologically active 1,25-dihydroxyvitamin D (1,25(OH)D) predominately in the kidneys. The adequate turnover of 1,25(OH)D is granted by the renal enzyme 24-hydroxylase (CYP24A1), which produces the inactive form 24,25-dihydroxyvitamin D (24,25(OH)D). 1,25(OH)D exerts its actions through interaction with the vitamin D receptor (VDR) which dimerizes, preferentially with retinoid X receptor (RXR). This complex binds to vitamin D response elements (VDREs) in the DNA of vitamin-D-sensitive genes to regulate transcription. Keyplayer genes of vitamin D metabolism are illustrated in blue with their respective genotyped polymorphisms in an illustrated box below. DHCR7, 7-dehydrocholesterol reductase; GC, group-specific component; CYP2R1, vitamin D-25-hydroxylase; 25(OH)D, 25-hydroxyvitamin D; CYP27B1, 25-hydroxyvitamin D3-1-alpha-hydroxylase; 1,25(OH)D, 1,25-dihydroxyvitamin D; CYP24A1, vitamin D3 24-hydroxylase; 24,25(OH)D, 24,25-dihydroxyvitamin D; VDR, vitamin D receptor; RXR, retinoid X receptor; VDRE, vitamin D response element; and mRNA, messenger RNA.

Based on recent publications, we selected a panel of genetic candidate targets that are known or suggested to influence 25(OH)D concentrations. As a further selection criterion, we defined a minor allele frequency (MAF) > 0.1. The selected genetic loci are located near genes that are involved in cholesterol synthesis, vitamin D transport, hydroxylation steps, VDRs and vitamin D degradation (Figure 1).

## 2. Experimental Section

### 2.1. Study Populations

All three RCTS were conducted independently as single-center, randomized, double-blind, placebo-controlled trials at the Medical University of Graz, Austria. In all three studies, an inclusion criterion among others was a 25(OH)D serum concentration below 75 nmol/l (divide by 2.496 to convert nmol/L to ng/mL), a threshold considered to define vitamin D insufficiency based on the guidelines of the Endocrine Society [24]. Participants were randomly allocated to the intervention or placebo (PBO) groups of the vitamin D RCTs. Methods and study design have been published in detail previously [25,26,27,28,29]. Briefly, study participants received 20,000 IU of cholecalciferol weekly equaling 50 oily drops per week or 7 oily drops per day (Oleovit D3© drops; Fresenius Kabi Austria) for 8 or 12 weeks, respectively. All subjects gave their informed consent for inclusion before they participated in these studies. These studies were conducted in accordance with the Declaration of Helsinki, and the protocol was approved by the Ethics Committee of the Medical University of Graz.

#### 2.1.1. “RCT1” Vitamin D, and Metabolic and Endocrine Parameters in Polycystic Ovary Syndrome (PCOS) Women and Healthy Controls

This trial was designed to investigate the effects of vitamin D supplementation on metabolic and endocrine parameters in women with polycystic ovary syndrome (PCOS, n = 180) according to the Rotterdam criteria [25] and in healthy premenopausal women (n = 150) [26]. To investigate short-time effects of vitamin D supplementation, a follow-up visit 12 weeks after inclusion into the study was scheduled. Data of this 12-week follow-up visit has been used for the present analyses. The trial was registered at clinicaltrials.gov (Clinical-Trials.gov Identifier NCT01721915) and at http://www.clinicaltrialregister.eu (EudraCT number, 2011-000994-30).

#### 2.1.2. “RCT2” The Graz Vitamin D and Total Testosterone (TT)-RCT

This RCT was designed to evaluate the effect of vitamin D supplementation over 12 weeks on serum testosterone (TT) levels in healthy men with low baseline serum TT concentrations (n = 100, TT < 10.4 nmol/l) [27] and in men with normal baseline serum TT concentrations (n = 100) [28]. The trial was registered at http://www.clinicaltrialsregister.eu (EudraCT number, 2011-003575-11) and at clinicaltrials.gov (ClinicalTrials.gov Identifier NCT01748370).

#### 2.1.3. “RCT3” The Styrian Vitamin D Hypertension Trial

This trial enrolled 200 participants with arterial hypertension (office blood pressure (BP) of systolic ≥140 mm Hg or diastolic ≥90 mm Hg, a mean 24-hour systolic ambulatory BP monitoring (ABPM) of systolic ≥125 mm Hg or diastolic ≥80 mm Hg, a home BP of systolic ≥130 mm Hg or diastolic ≥85 mmHg, or ongoing antihypertensive treatment). This RCT investigated whether vitamin D supplementation lowers ABPM values and improves cardiovascular risk factors [29]. The trial was initially registered at http://www.clinicaltrialsregister.eu (EudraCT number, 2009-018125-70) and was additionally registered at clinicaltrials.gov (ClinicalTrials.gov Identifier NCT02136771).

The inclusion criteria for the present investigation were completion of the vitamin D RCTs and allocation to the intervention group of vitamin D which were for RCT1: 218, for RCT2: 100 and for RCT 3: 100 participants.

### 2.2. Procedures

Body weight and height were measured in all participants. Basal blood samples for laboratory parameters were collected between 7.00 and 9.00 a.m. after an overnight fast. 25(OH)D measured by immunoassays was used for evaluation of inclusion criteria. Serum 25(OH)D levels were additionally measured by adjusted isotope-dilution liquid chromatography-tandem mass spectrometry (ID-LC-MS/MS) methods in 2018 [30]. 25(OH)D levels measured by ID-LC-MS/MS were used for statistical analyses. Change in ∆25(OH)D was calculated as serum 25(OH)D levels (nmol/l) after 8 weeks (RCT3) or 12 weeks (RCT1 and RCT2) minus serum 25(OH)D levels at baseline (nmol/l) after inclusion into the study.

### 2.3. SNP Selection and Genotyping

Ten single nucleotide polymorphisms (SNPs) out of 6 genes were selected according to loci with influence on 25(OH)D levels according to published data or a reported association to diseases in relation to vitamin D. Based on a literature search, a panel of genetic candidate targets was selected by having an effect on vitamin D levels. As further selection criterion, we defined a minor allele frequency (MAF) > 0.1. (Table 1). The selected loci are located near genes that are involved in cholesterol synthesis, vitamin D transport, hydroxylation steps, vitamin D receptors (VDRs) and vitamin D degradation.

Genomic DNA was prepared from ethylenediaminetetraacetic acid (EDTA) anticoagulated peripheral mononuclear blood cells (PBMCs) by using a common salting-out procedure. Genotypes were determined by fluorogenic 5′-exonuclease assays (TaqMan™). Primer and probe sets except for CYP27B1 (rs703842) were designed and manufactured as assays on demand by Applied Biosystems (Life Tech Austria, Vienna, Austria). Assay IDs of all SNPs are shown in Table 1. CYP27B1 rs703842 was not available as an assay on demand. Primer and probe sets were therefore custom designed and manufactured using Applied Biosystems “Assay-by-Design” custom service (Life Tech Austria, Vienna, Austria). Endpoint fluorescence was measured in a Fluoroskan Ascent plate reader (Thermo Labsystems, Helsinki, Finland). Fluorescence data were exported into excel format and analyzed as scatter plots.

### 2.4. Statistics

Statistical analysis was done using SPSS statistics version 25 (IBM Deutschland GmbH, Ehningen, Germany). Distribution of data was analysed by descriptive statistics and the Kolmogorov–Smirnov test. Continuous data with a normal distribution are presented as mean ± standard deviation and were compared between genotype groups by univariate analysis of variance (ANOVA). Seasonal variations of vitamin D concentrations were modeled using categorial variables for the month of blood sampling (season 1: October till March, season 2: April till June and season 3: July till September). We performed adjustment for season and BMI as well as an unadjusted model. The Chi-squared test was used to compare genotype frequencies between ∆25(OH)D subgroups and to determine deviations from the Hardy–Weinberg equilibrium. A *p*-value < 0.05 was considered statistically significant. 25(OH)D subgroups has have been categorized by tertiles of the ∆25(OH)D range of all patients (low ∆25(OH)D increase: −42 to <26 nmol/l, mean ∆25(OH)D increase: 26 to <94 nmol/l and high ∆25(OH)D increase: 94 to 163 nmol/l).

## 3. Results

**Figure 2:**
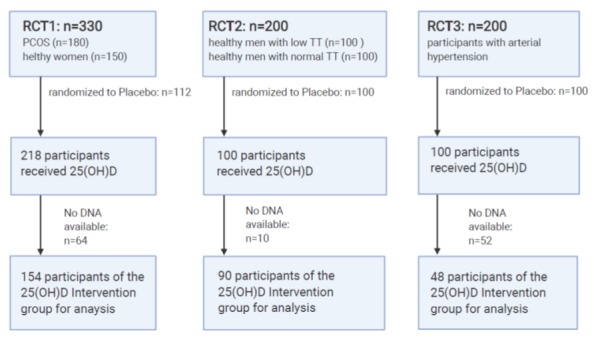
Study flow chart. RCT, randomized controlled trial; TT, testosterone; PCOS, polycystic ovary syndrome; n, number; DNA, deoxyribonucleic acid.

We included 260 women (63.3%) and 151 (36.7%) men in our analyses. Demographic data of the study population are given in Table 2 for each RCT. Genotypes were successfully determined in 142 (65.1%) participants of RCT1, in 90 (90.0%) participants of RCT2 and in 41 (44.1%) of RCT3. No DNA samples were available from the remaining subjects. A study flow chart is given in Figure 2.

Genotype frequencies of all 10 SNPs did not deviate from the Hardy–Weinberg equilibrium in the entire study population of genotyped participants.

Genotypes and ∆ 25(OH)D levels are shown in Table 3. The minor A-allele of the VDR rs10783219 polymorphism was significantly associated with lower ∆25(OH)D values in the entire study population (*p* = 0.022). This result was reflected as a trend in the RCT2 cohort (*p* = 0.083) but was not replicated in RCT1 or RCT3 when analyzed separately (Table 3).

Unadjusted application of ANOVA showed significant associations of VDR rs10783219 and ∆25(OH)D in the entire study population (*p* = 0.013) as well as in RCT2 (*p* = 0.045) and as a trend in RCT 3 (*p* = 0.079).

Nevertheless, ∆25(OH)D levels did not rise or fall according to a typical gene dose effect. In our investigation, heterozygous VDR rs10783219 genotypes displayed the highest increase in ∆25 (OH)D levels (Table 3).

Vitamin D subgroups have been categorized according to tertiles of the total range of all participants. Genotype frequencies of all polymorphisms did not show any statistically different distribution between ∆25(OH)D subgroups (low ∆25(OH)D increase: −42 to <26 nmol/l, mean ∆25(OH)D increase: 26 to <94 nmol/l and high ∆25(OH)D increase: 94 to 163 nmol/l).

## 4. Discussion

In the present study, we present data of 10 polymorphisms out of 6 genes analysed in 411 participants with 25(OH)D levels < 75 nmol/l participating in vitamin D RCTs who were allocated to the intervention group. We identified VDR rs10783219 as a genetic variant associated with lower ∆25(OH)D levels after vitamin D supplementation. As an intronic SNP, VDR rs10783219 may have unexpected deleterious effects on the splicing of the gene transcript. This may alter the dimerization of VDR with retinoid X receptor (RXR) and or the binding of the ligand-activated transcription factor to vitamin-D-responsive elements in promoter regions of target or regulatory genes. The exact regulatory mechanism of how this may decelerate the 25(OH)D increase still needs to be elucidated. This association was present in all 3 RCTs analysed together as well as a trend in RCT2, calculated separately. This finding reemphasizes the relevance of VDR genetics not only concerning 25(OH)D levels but also regarding vitamin D increase after supplementation.

As expected, we observed large variations between serum concentrations of 25(OH)D that are in general explained by environmental factors such as latitude, season, cultural issues, diet and intestinal factors or fortified food policies. Furthermore, individual sociocultural and other factors such as age, obesity, clothing, the use of sunscreen, sunbathing habits, the use of vitamin supplements or skin pigmentation are intensely affecting vitamin D status [1,39,40]. During the summer, these environmental factors are the prevailing mechanisms influencing 25(OH)D levels, whereas during the winter season when 25(OH)D levels are at their lowest, serum concentrations of 25(OH)D are under considerable genetic influence [41]. The VDR rs10783219 polymorphism might therefore be relevant during the months of October to March when hardly any endogenous vitamin D can be generated.

Nevertheless, ∆25(OH)D levels in our investigation did not rise or fall according to a typical gene dose effect, which describes the relatively linear relationship between a phenotypic value and the contributing genes among codominant alleles. In our investigation, heterozygous VDR rs10783219 genotypes displayed the highest increase in ∆25 (OH)D levels. Therefore, we cannot rule out a possible bias of unknown source and have to be aware not to overinterpret this finding.

Further, we have decided deliberately to not correct the *p*-value for multiple testing as our SNPs were all carefully selected based on a prior data suggesting an effect on 25(OH)D status. Correcting for multiple testing, e.g., using Bonferroni correction, reduces the rate of false positive findings but at the same time reduces statistical power and increases the risk of false negative results [42]. We therefore did not apply Bonferroni correction but strongly encourage independent replication of our results in further studies [43].

Although there are several reports on an effect of genetic variants on basal serum 25(OH)D levels [11,12], only few studies have reported investigations on vitamin D supplementation [21,44,45,46], leaving a potential role of genetic polymorphisms on 25(OH)D increase open. In the few papers describing these outcomes, the authors found inconsistent effects of genetic variants on 25(OH)D increase.

Didriksen et al. [21] observed in their combined investigation a ∆25(OH)D of −5.8 nmol/l between the major and the minor homozygote CYP2R1 rs10741657 genotypes after 6 months of a 40,000 IU per week supplementation. Even though Didriksen et al. used a higher vitamin D dose (40,000 IU per week vs. 20,000 IU per week in our RCT), the increase of 25(OH)D seems comparable to our results. In our cohort, the ∆25(OH)D difference between the major and the minor homozygotes of CYP2R1 rs10741657 genotypes was −3.8 nmol/l after 8 and 12 weeks, respectively, of supplementation. This difference was not statistically significant in our cohorts.

Fu et al. [44] investigated 98 healthy adults in a 1-year vitamin D intervention study with 4200 IU/week or with 28,000 IU/ week and concluded that healthy participants with different GC rs4588 genotypes have different responses to the same 25(OH)D dose. Likewise, Nimithphong et al. reported associations of this polymorphism on responsiveness to vitamin D3 (but not D2) after vitamin D3 and vitamin D2 supplementation in a relatively small cohort (*n* = 39) [45].

In contrast, genotypes of GC rs4588 were not related to the increase of vitamin D in our data set, which is in line with Waterhouse et al. [46], who did not find an association between GC rs4588 and ∆25(OH)D in 350 Australians between 60 and 84 years who received 15,000 IU or 75,00 IU 25(OH)D for 12 months, respectively.

Inconsistencies in the current literature might be, at least in part, explained by the differences in study designs and duration as well as the broad variations in study population sizes. In addition, vitamin D doses varied with regard to quantity and frequency (e.g., daily vs. weekly vs. monthly supplementation), potentially affecting the comparability of some studies.

Metabolic differences in vitamin D metabolism due to genetic variations of GC, CYP2R1, CYP27B1 and CYP24A1 genes likely affect serum vitamin D derived from endogenous production or given as a supplement in a similar matter. This might be different for polymorphisms in the DHCR7 gene, which mainly affect UV-induced vitamin D synthesis. Therefore, these polymorphisms may not be as relevant for an increase of vitamin D after supplementation as polymorphisms in other genes contributing to vitamin D metabolism.

Finally, Seuter et al. [47] evaluated an interesting multiple parameter determination of the vitamin D responsiveness without regard of genetic influence. The correlation analysis is based on changes in parathyroid hormone (PTH) serum concentrations after ingestion of a single oral dose of vitamin D 800,000 IU and resultant vitamin D modulated chromatin accessibility. This allows for a classification into high, mid and low vitamin D responders in healthy young individuals, which does not reflect the actual 25(OH)D serum levels but the inducibility by the hormone.

Our study has some limitations. First, DNA was not provided from all participants; especially in RCT3, only 44% of the study participants signed an informed consent for genetic testing. Further, our data are limited by the fact that we investigated vitamin D insufficiency in specific cohorts of women with polycystic ovary syndrome, of men with low TT levels, of participants with arterial hypertension as well as of healthy men and women with baseline 25(OH)D levels <75 nmol/l. Our results may therefore not be generalizable to the general population or other diseases. Second, the investigated VDR rs10783219 may not be the causal variant but might be in linkage disequilibrium with a potential important locus. Other yet unknown SNPs within the VDR gene may also be associated with vitamin D increase after supplementation.

Further, there is no clear recommendation to perform follow-up measurements of serum 25(OH)D after starting vitamin D supplementation. In our investigation, we compared data of vitamin D RCTs after 8 weeks (RCT3), which is approximately the time required to reach a steady state according to some studies [48,49], whereas others indicate that it may take even 12 weeks to obtain stable serum levels [50]. Finally, the size of the present study was limited for a genetic association study. Therefore, our results require replications in other vitamin D supplementation cohorts. We did not evaluate possible associations between SNP genotypes and vitamin D metabolite ratios, which may be considered as a further limitation.

Strengths of our investigation are the in-depth clinical and biochemical characterization of all patients as well as the use of state-of-the-art and standardized liquid chromatography–mass spectrometry (LCMS) method to measure 25(OH)D concentrations in all samples and the randomized controlled design of the studies.

In summary, we investigated a selected panel of allelic determinants with a reported association to 25(OH)D levels and their influence on vitamin D increases after supplementation, showing a significant association of a VDR variant with ∆25(OH)D levels. VDR rs10783219 might therefore, if replicated in further RCTs, be a new aspect taken into account when discussing individual vitamin D supplementation requirements.

## Figures and Tables

**Table 1:** Selected single nucleotide polymorphisms.

Gen	SNPs	AOD	Literature
GC	rs7041	C___3133594_30	Wang et al. 2010 [11]
			Ahn et al. 2010 [31]
GC	rs4588	C___8278879_10	Trummer et al. 2012 [32]
GC	rs1155563	C___8278782_20	Pena-Chilet et al. 2013 [33]
GC	rs12512631	C___3133604_10	Nissen et al. 2014 [34]
CYP27B1	rs703842	Custom	Orton et al. 2008 [8]
			McGrath et al. 2010 [35]
			Jiang et al. 2016 [36]
DHCR7	rs12785878	C__32063037_10	Trummer et al. 2013[6]
			Wang et al. 2010 [11]
CYP2R1	rs10741657	C___2958430_10	Wang et al. 2010 [11]
			Trummer et al. 2013 [6]
			Nissen et al. 2014; [34]
CYP24A1	rs6013897	C__29958084_10	Wang et al. 2010; [11]
VDR	rs2228570	C__12060045_20	Mokry et al. 2015 [37]
VDR	rs10783219	C___2880803_10	Barry et al. 2014 [38]

Genes, rs numbers, assay on demand (AOD) numbers and source of literature of each selected genetic variant. GC, group specific component; CYP27B1, 1-alpha-hydroxylase; DHCR 7,7-dehydrocholesterol reductase, CYP2R1, vitamin D-25-hydroxylase; CYP24A1, vitamin D3 24-hydroxylase; VDR, vitamin D receptor; SNP, single nucleotide polymorphism.

**Table 2:** Demographic data of the study.

	RCT1		RCT2		RCT3	Total
	Vitamin D, and Metabolic and Endocrine Parameters		Vitamin D and TT in Men		Styrian Vitamin D and Hypertension Trial	All RCTs Combined
	Women with PCOS	Healthy Women	Men with Low TT Levels (<10.4 nmol/l)	Healthy Men	Participants with Arterial Hypertension	
*n*	119 (180)	99 (150)	50 (100)	50 (100)	93 (200)	411 (730)
Sex male	n.a.	n.a.	50	50	51 (55%)	151
Sex female	119 (100%)	99 (100%)	n.a.	n.a.	42 (45%)	260
Age (yr)	25.7 ± 0.5	35.7 ± 1.0	46.9 ± 1.6	38.0 ± 1.8	60.8 ± 1.2	65.8 ± 1.2
BMI (kg/m2)	26.7 ± 0.7	25.7 ± 0.6	29.3 ± 0.6	24.9 ± 0.5	30.4 ± 0.5	27.4 ± 0.6
25(OH)D at screening (nmol/l)	48.1 ± 1.9	55.4 ± 2.1	56.8 ± 2.5	52.5 ± 2.2	46.3 ± 1.5	51.8 ± 2.0
25(OH)D after 8 weeks of supplementation (nmol/l)	n.a.	n.a.	n.a.	n.a.	61.4 ± 2.0	61.4 ± 2.0
25(OH)D after 12 weeks of supplementation (nmol/l)	92.2 ± 2.7	92.8 ± 2.7	98.1 ± 4.1	106.2 ± 3.1	n.a.	97.3 ± 3.2

Demographic data of participants allocated to the vitamin D group within RCT1, 2 and 3: Frequency data are presented as number (number of RCT participants in total), (percentage), continuous data as mean ± standard deviation. n.a. data not available. RCT, randomized controlled trial; TT, testosterone; PCOS, polycystic ovary syndrome.

**Table 3:** Delta 25(OH)D values according to selected SNP genotypes.

		RCT1 (*n* = 218)		RCT2 (*n* = 100)		RCT3 (*n* = 93)		All (*n* = 411)	
		Vitamin D, and Metabolic and Endocrine Parameters		Vitamin D and TT in Men		Styrian Vitamin D and Hypertension Trial		All	
SNP		Delta 25(OH)D (nmol/l)	*p*-Value	Delta 25(OH)D (nmol/l)	*p*-Value	Delta 25(OH)D (nmol/l)	*p*-Value	Delta 25(OH)D (nmol/l)	*p*-Value
GC_rs7041	AA	30.8 ± 4.7		55.1 ± 8.5		7.5 ± 8.6		30.5 ± 4.6	
	AC	40.7 ± 3.0	0.601	45.3 ± 4.7	0.216	10.6 ± 4.5	0.499	37.5 ± 2.5	0.423
	CC	36.0 ± 5.8		53.5 ± 6.9		6.9 ± 3.9		34.2 ± 4.7	
GC_rs4588	GG	38.6 ± 4.0		51.7 ± 4.4		−1 ± 4.1		37.0 ± 3.1	
	GT	39.0 ± 3.1	0.233	46.0 ± 6.1	0.267	8.0 ± 4.8	0.765	35.6 ± 2.8	0.532
	TT	24.9 ± 6.6		49.8 ± 15.9		4.2 ± 11.8		23.4 ± 7.2	
GC_rs12512631	CC	38.0 ± 8.0		37.4 ± 6.0		−19.0 ± 3.0		32.6 ± 6.0	
	CT	39.5 ± 3.4	0.765	51.4 ± 4.5	0.323	5.9 ± 3.5	0.859	39.4 ± 2.7	0.149
	TT	36.1 ± 3.6		50.1 ± 7.0		5.5 ± 4.8		32.0 ± 3.2	
GC_rs1155563	CC	31.0 ± 5.8		36.9 ± 11.0		8.4 ± 13.5		26.8 ± 6.6	
	CT	38.8 ± 3.0	0.417	48.6 ± 8.0	0.371	10.0 ± 6.0	0.924	36.4 ± 3.0	0.774
	TT	37.7 ± 4.1		51.9 ± 3.5		−0.8 ± 3.6		36.3 ± 3.0	
DHCR7_rs12785878	GG	36.6 ± 4.3		38.0 ± 5.0		−6.0 ± 18.9		31.9 ± 4.7	
	GT	37.3 ± 4.3	0.866	43.2 ± 4.0	0.114	7.8 ± 5.1	0.557	32.6 ± 2.9	0.220
	TT	39.2 ± 3.5		55.3 ± 5.8		5.9 ± 4.5		40.2 ± 3.2	
CYP24A1_rs6013897	AA	32.5 ± 8.3		50.0 ± 6.0		1.0 ± 0.0		32.8 ± 7.1	
	AT	39.9 ± 3.6	0.944	54.2 ± 8.5	0.633	−1.5 ± 5.3	0.334	36.0 ± 4.0	0.890
	TT	37.7 ± 3.2		47.0 ± 3.7		9.0 ± 4.5		35.3 ± 2.4	
VDR_rs2228570	AA	36.2 ± 6.3		41.3 ± 6.8		13.8 ± 16.6		35.3 ± 4.6	
	AG	37.7 ± 3.4	0.933	51.3 ± 5.2	0.532	−0.6 ± 4.8	0.806	36.1 ± 3.0	0.957
	GG	39.3 ± 3.8		50.6 ± 6.5		9.3 ± 4.8		35.2 ± 3.2	
VDR_rs10783219	AA	36.4 ± 3.5		44.3 ± 4.5		−4.9 ± 4.8		31.0 ± 3.0	
	AT	41.8 ± 3.5	0.458	58.2 ± 6.6	0.083	13.2 ± 4.3	0.153	41.4 ± 3.1	0.022
	TT	27.9 ± 7.4		38.7 ± 4.4		7.0 ± 9.5		29.7 ± 4.5	
CYP27B1_rs70384	CC	30.5 ± 7.4		52.4 ± 12.3		−0.8 ± 19.5		32.5 ± 7.0	
	CT	35.2 ± 3.8	0.147	47.5 ± 3.7	0.288	7.7 ± 5.0	0.322	35.0 ± 2.7	0.514
	TT	42.7 ± 3.1		52.5 ± 9.9		4.1 ± 4.3		36.8 ± 3.3	
CYP2R1_rs10741657	AA	34.9 ± 5.9		56.7 ± 10.6		−6.5 ± 9.2		33.8 ± 5.5	
	AG	40.9 ± 3.4	0.417	45.0 ± 3.8	0.422	1.3 ± 5.6	0.982	35.4 ± 2.8	0.822
	GG	35.3 ± 3.8		53.1 ± 7.2		12.1 ± 5.5		37.6 ± 3.6	

Genotypes and ∆ 25(OH)D levels in RCT1, 2 and 3: ∆25(OH)D levels are shown according to SNP genotypes: GC_rs7041, GC_rs4588, GC_rs12512631, GC_rs1155563, DHCR7_rs12785878, CYP24A1_rs6013897, VDR_rs2228570, VDR_rs10783219, CYP27B1_rs70384 and CYP2R1_rs10741657. 25(OH)D values in nmol/l are presented as mean ± standard deviation. *p*-values were calculated by ANOVA for genotype group differences between ∆25(OH)D values with adjustment for season of blood drawing and BMI. RCT, randomized controlled trial; TT, total testosterone; 25(OH), 25-hydroxyvitamin D; GC, group-specific component; DHCR7, 7-dehydrocholesterol reductase; CYP24A1, vitamin D3 24-hydroxylase; VDR, vitamin D receptor; CYP27B1, 25-hydroxyvitamin D3-1-alpha-hydroxylase; CYP2R1, vitamin D-25-hydroxylase; and BMI, body mass index.
